# Synthesis of New Cisplatin Derivatives from Bile Acids

**DOI:** 10.3390/molecules25030655

**Published:** 2020-02-04

**Authors:** Barbara Seroka, Zenon Łotowski, Agnieszka Hryniewicka, Lucie Rárová, Rafal R. Sicinski, Aneta M. Tomkiel, Jacek W. Morzycki

**Affiliations:** 1 Faculty of Chemistry, University of Białystok, K. Ciołkowskiego 1K, 15-245 Białystok, Poland; aga_h@uwb.edu.pl (A.H.); a.tomkiel@uwb.edu.pl (A.M.T.); morzycki@uwb.edu.pl (J.W.M.); 2 Laboratory of Growth Regulators, Faculty of Science, Palacký University, and Institute of Experimental Botany of the Czech Academy of Sciences, Šlechtitelů 27, CZ-78371 Olomouc, Czech Republic; lucie.rarova@upol.cz; 3 Faculty of Chemistry, University of Warsaw, Pasteura 1, 02-093 Warsaw, Poland

**Keywords:** bile acids, lithocholic acid, deoxycholic acid, hyodeoxycholic acid, cholic acid, steroidal diamines, platinum(II) complexes

## Abstract

A series of bile acid derived 1,2- and 1,3-diamines as well as their platinum(II) complexes were designed and synthesized in hope to get a highly cytotoxic compound by the combination of two bioactive moieties. All complexes obtained were subjected to cytotoxicity assays in vitro and some hybrid molecules showed an expected activity.

## 1. Introduction 

**Figure 1:**
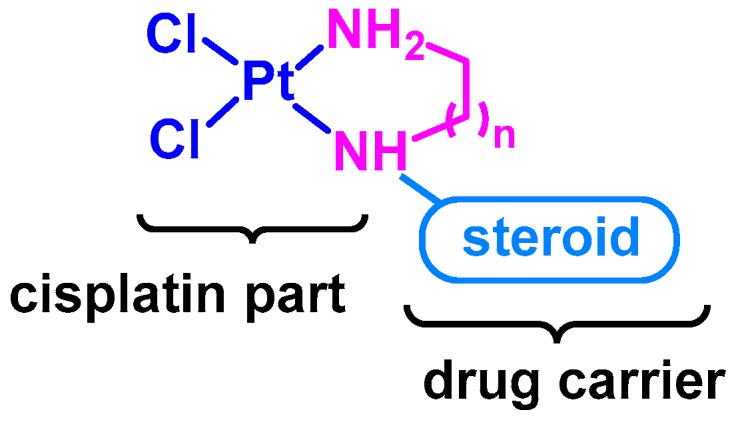
General model of steroid-based cisplatin derivatives (n = 1 or 2).

Cisplatin is one of the most effective chemotherapeutic agents used in the treatment of many human cancers [1]. Unfortunately, its therapeutic use is limited due to harmful side effects, in particular nephrotoxicity [2,3]. Although many anticancer platinum complexes have been developed so far [4], there is still a need to find more active and more selective drugs. Combining of two bioactive molecules as a way to improve the biological properties of the starting compounds is a common practice in medical chemistry [5]. In addition, a natural compound that is designed to form a hybrid molecule together with cisplatin should be a good transporter for transferring the drug to cancer cells. Binding biomolecules to drugs always causes a drastic change in the structure of the carrier as well as in the structure of the pharmacologically active compound. We recently used this idea to design steroid-based cisplatin derivatives according to the general formula shown in Figure 1 [6,7].

**Figure 2:**
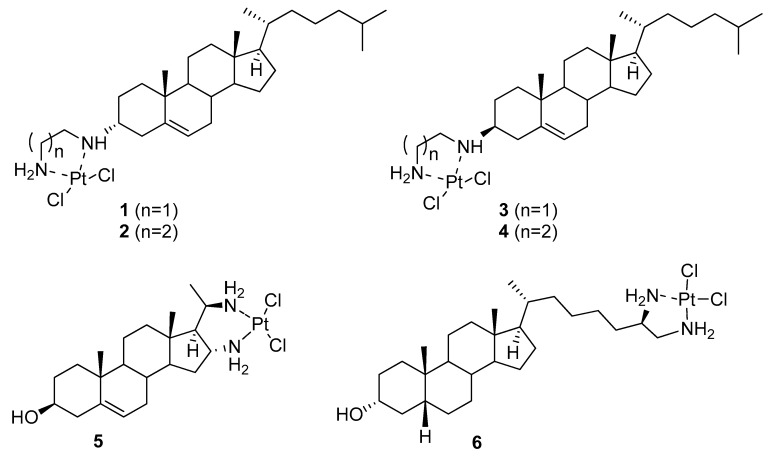
Steroid-based cisplatin derivatives [6,7].

As a result of our previous work, steroidal cisplatin analogs were obtained: two series of cholestane-based Pt(II) complexes derived from cholesterol (**1**–**4**) and a complex **5** (Figure 2) synthesized from 16-dehydropregnenolone acetate [6]. However, the most promising was cisplatin derivative **6** based on lithocholic acid (LCA) [7]. Although the complex **6** showed only moderate cytotoxicity (IC_50_ 28 µM) in the initial test against the HeLa cervical cancer cell line, it proved to be non-toxic to normal fibroblasts. This selectivity opens the way for modification of this complex to be more active while maintaining a lack of toxicity to healthy cells.

The biological functions of bile acids depend on their chemical nature and polarity [8]. Both LCA and deoxycholic acid (DCA) are hydrophobic but show different toxicity [9]. Bajaj et al. [10] have reported that DCA having two hydroxyl groups shows weak interactions with cell membranes compared to highly hydrophobic LCA. The hydration barrier between cell membranes and DCA does not favor the interactions that cause poor uptake in mammalian cells. In contrast, more hydrophobic LCA can easily penetrate cell membranes. In addition, the varied activity of DCA and LCA may also result from their different binding to cellular receptors [11,12,13]. Lithocholic acid exhibits high antitumor activity [14,15], is involved in human and animal carcinogenesis [16], can selectively kill neuroblastoma cell lines [17], and selectively induces apoptosis in prostate [18] and colon cancer cells [19]. LCA elicits apoptosis by activation both extrinsic and intrinsic pathways without entering to cancer cells. It has been suggested that target of cancerous tissue is most likely localized at the surface of the plasma membrane and is either activated or deactivated by LCA in order to elicit apoptosis [18]. 

Bile acids directly activate various intracellular ligand-activated nuclear receptors, such as the farnesoid X receptor (FXR), pregnane X receptor (PXR), and vitamin D receptor (VDR), and cell surface G protein-coupled receptors (GPCRs), such as the G protein-coupled bile acid receptor (TGR5 and Gpbar-1). FXR and PXR are highly expressed in tissues that are exposed to bile acids, including the liver and the intestine, whereas VDR is widely expressed in most tissues. TGR5 is highly expressed along the intestinal tract, with the highest expression found in the ileum and colon. It is also expressed in nontraditional bile acid target organs including white and brown adipose, spleen, kidney, pancreas, lung, macrophages, and the central nervous system [20]. Moreover, some receptors found to be overexpressed in cancer cell lines, e.g., GPCRs are overexpressed in lung, prostate, breast, and neuroblastomas. This fact is exploited in targeting drugs to cancerous tissues and the tumor microenvironment [21]. For this reason, bile acids have become important carriers of drugs in medicinal chemistry [22]. Furthermore, the combination of a pharmacologically active compound with bile acid makes it possible to exploit the natural resorption and can lead to better uptake of pharmaceuticals [23]. Paschke et al. have reported synthesis and biological evaluation of Pt(II)-based cholic acid derivatives, which consist of three functional parts—a transport fragment, a spacer and a biologically active drug component: cisplatin [24] or carboplatin [25]. Some other anticancer Pt(II)-based bile acid derivatives are known, e.g. Bamet-R1, Bamet-R2, Bamet-UD1, or Bamet-UD2, in which platinum is bound to the glycocholic or ursodeoxycholic acids [26,27,28,29,30]. Bartoli et al. have reported another example of Pt(II) complex of cholic acid, in which functional groups present in the steroidal A ring coordinate with platinum [31]. All the above-mentioned complexes contain an ester linkage between the steroid moiety and platinum-based drug, which guarantees the release of the cisplatin-like metabolite. The complex **6** is, to the best of our knowledge, the first Pt(II) complex with stable linkage of LCA and cisplatin, based on carbon–carbon bond. This approach ensures that LCA is capable of playing double role: as a carrier and anticancer co-drug. A spacer between cisplatin and biomoiety is needed because it separates the active parts of both molecules so that they can be recognized and act independently. Such an arrangement assures that their biological properties are preserved [32] and also some synergic effects can be expected.

Taking into account the promising cytotoxic properties of the cisplatin analog **6** [7], as well as the chemical nature of bile acids, we designed new hybrid platinum moieties. We synthesized two series of platinum complexes with a different distance between the amino groups based on four bile acids: three hydrophobic (LCA, DCA, hyodeoxycholic) and one hydrophilic (cholic acid). The goal of our research is to find the most active and most selective compound as well as to compare the activity of different bile acid derivatives. 

## 2. Results and Discussion

### 2.1. Chemistry

**Scheme 1:**
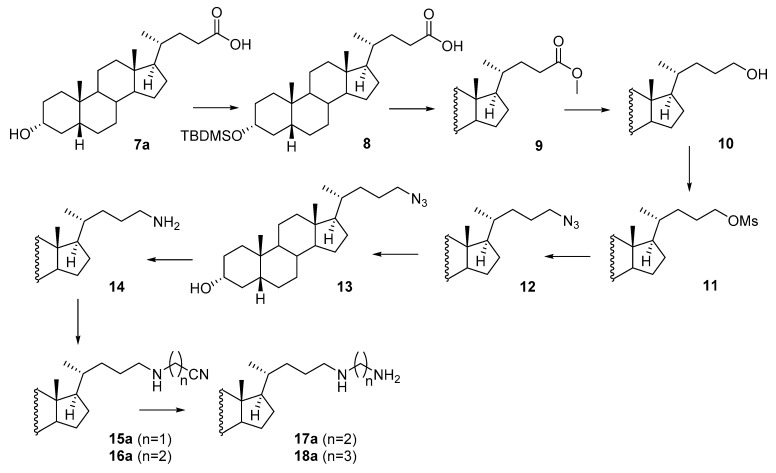
Synthesis of 1,2- and 1,3-diamines from lithocholic acid (LCA).

First attempts to synthesize steroidal analogs of cisplatin employed the synthetic paths that we previously developed [6]. The strategy for the 1,2-diamine and 1,3-diamine synthesis from commercially available LCA (**7a**) is shown in Scheme 1. Firstly, the hydroxyl group at C-3 was protected as TBDMS ether (**8**). For the conversion of carboxylic acid to corresponding amine, and then to 1,2- and 1,3-diamines, the multi-step transformation of the side chain was carried out. The methylation of the carboxylic group with methanol/HCl was followed by LiAlH_4_ reduction of methyl ester **9** to primary alcohol **10**. Then the hydroxyl group at C-24 was converted into methanesulfonate **11**, which was further transformed into azide **12** with sodium azide in DMF [33]. Deprotection of the hydroxyl group at C-3 allowed to obtain **13** in a good overall yield (all steps were fairly efficient). The Staudinger reaction of azide **13** with triphenylphosphine yielded aminosteroid **14**. In the next step an introduction of 2-cyanoethyl or cyanomethyl chains to **14** was attempted. This would allow for an extension of the side chain with simultaneous introduction of the second nitrogen atom. The alkylation of **14** with bromoacetonitrile in the presence of Na_2_CO_3_ in ethanol afforded 24-(*N*-cyanomethyl)-aminocholan-3α-ol (**15a**). Alternatively, **14** reacted with acrylonitrile to afford the addition product—24-(*N*-2′-cyanoethyl)-aminocholan-3α-ol (**16a**). The final step of the synthesis was the reduction of nitrile functional groups with lithium aluminum hydride to give products **17a** and **18a** in high yields. 

The synthesis allowed to obtain 24-(2′-amino-ethylamino)-cholan-3α-ol (**17a**) and 24-(3′-amino-propylamino)-cholan-3α-ol (**18a**) in satisfactory total yields. However, a large number of steps due to necessary protection of functional groups prompted us to look for a shorter method of synthesis of these compounds.

**Scheme 2:**
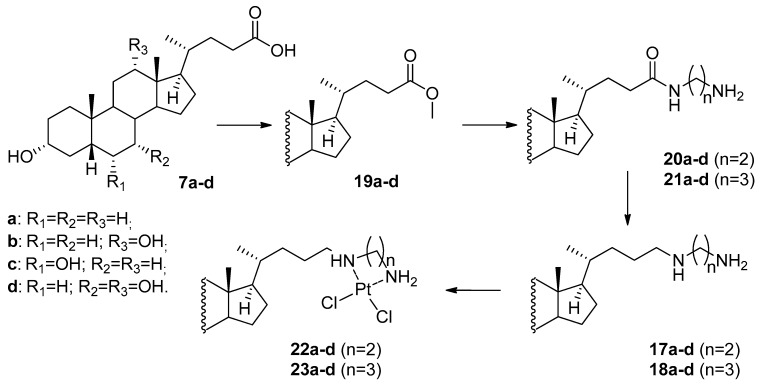
Synthesis of cisplatin derivatives from different bile acids.

Starting materials for an alternative synthesis of these bidentate ligands were bile acids, namely lithocholic acid (**7a**), deoxycholic acid (**7b**), hyodeoxycholic acid (**7c**), and cholic acid (**7d**). The structures of these compounds along with their designed modifications are shown in Scheme 2 and Table 1. 

The aim of these chemical transformations of bile acids was to introduce a strongly chelating group into the side chain of steroid. Since amines are ligands of choice for transition metals, a series of eight steroidal diamines derived from four different bile acids were designed. The two amino groups placed at the side chain terminal should be relatively close to each other to make sure that the configuration at the platinum atom in the complex is *cis* (it is known that the *trans* isomers are biologically inactive). In the first step of synthesis the carboxylic group was activated as a methyl ester by treatment of bile acids **7a**–**d** with methanol in the presence of catalytic amounts of concentrated hydrochloric acid. At the second step, esters **19a**–**d** were subjected to aminolysis with an excess of ethylenediamine or propane-1,3-diamine in methanolic solution. The reactions were rather slow—they were completed at 65 °C in four days. In this way, two series of amides were obtained in high (about 90%) yields: **20a**–**d** and **21a**–**d**, respectively. The last step of the ligand synthesis consisted of the reduction of the amides with lithium aluminum hydride under relatively harsh conditions (3 days at reflux in THF). Since the obtained diamines strongly bound to aluminum ions, a special procedure was elaborated for the product isolation. The method consisted of treatment of the reaction mixture with a small amount of aqueous sodium hydroxide. Then water was removed with anhydrous sodium sulfate and all inorganic material was filtered off. The products in both series, 1,2-diamines (**17a**–**d**) and 1,3-diamines (**18a**–**d**), were obtained in satisfactory (62–73%) yields. These steroidal ligands were used in the next step for the complexation reaction with potassium tetrachloroplatinate according to the known procedure [34]. The platinum complexes **22a**–**d** and **23a**–**d** were characterized by IR, MS and ^195^Pt NMR spectra. The IR spectra of complexes displayed sharp bands in the range 3223–3091 cm^−1^ assigned to N-H stretching characteristic for coordination. The mass spectra showed a characteristic pattern resulting from platinum isotope distribution (i.e., ^194^Pt, ^195^Pt and ^196^Pt). Considering that ^195^Pt yields relatively narrow signals and has a wide chemical shift range, the recorded NMR spectra of platinum complexes confirm both their purity and structure. The observed chemical shifts of ^195^Pt signals in the spectra of the synthesized complexes **22a**–**d** and **23a**–**d** lie within the range characteristic for complexes of the type PtN_2_Cl_2_. Thus, the chemical shifts of the former compounds are very similar to these of substituted ethylenediamine complexes (ca. −2360 ppm) reported in the literature [35] whereas δ values of the latter resemble this found [36] in the *cis*-Pt(MeNH_2_)_2_Cl_2_ complex (−2222 ppm). 

### 2.2. Biological Evaluation

All new platinum(II) complexes were screened for cytotoxic activity against following tumor cell lines: T-lymphoblastic leukemia (CEM), epithelial breast carcinoma (MCF7), and cervical carcinoma cell lines (HeLa) (Table 2). A control of cytotoxicity was done with normal human fibroblasts (BJ) and human umbilical vein endothelial cells (HUVEC). The cytotoxicity of all substances in BJ and HUVEC was very similar. Lithocholic acid-based complex **23a** proved to be the most active among all tested compounds. Its sensitivity against HeLa cell line (7.0 µM) was better than that of cisplatin (11.4 µM). However, the toxicity of **23a** against normal human cells (BJ and HUVEC) was similar to that of cisplatin. It should be noticed that lithocholic acid-based complex **22a** and deoxycholic acid-based derivatives: **22b** and **23b** showed a moderate cytotoxic activity. This result is consistent with that obtained for complex **6** [7]. Taking into account lack of activity of cholic and hyodeoxycholic acids-based complexes at studied concentrations (against all tested cell lines IC_50_ > 50 µM) we can suppose that modifications of bile acids should be focused on the most lipophilic lithocholic acid derivatives. The results presented here provide an inspiration for further studies in this area. 

## 3. Materials and Methods

### 3.1. General

NMR spectra were recorded with an Avance II 400 spectrometer (Bruker, Fällanden, Switzerland) operating at 400 MHz (^1^H), 100 MHz (^13^C) or 86 MHz (^195^Pt) using CDCl_3_ and CD_3_OD solutions (with TMS as the internal standard) or DMF in case of Pt(II) complexes. Infrared spectra were recorded using Attenuated Total Reflectance (ATR) as solid samples with a Series II Magna-IR 550 FT-IR spectrometer (Nicolet, Madison, WI, USA). Mass spectra were obtained at 70 eV with an AMD-604 spectrometer (Agilent, Santa Clara, NJ, USA) and Accurate-Mass Q-TOF LC/MS 6530 spectrometers with electrospray ionization (ESI). The reaction products were isolated by column chromatography, performed using 70–230 mesh silica gel (J.T. Baker, Phillipsburg, NJ, USA). Bile acids were purchased from TCI EUROPE N.V. Compound **13** was synthesized according to literature procedure [33] starting from lithocholic acid (**7a**) in 77% total yield.

### 3.2. Chemical Synthesis

#### 3.2.1. Reduction of 24-azidocholan-3α-ol (**13**)

To a solution of azide **13** (0.49 g, 1.27 mmol) in THF (20 mL), triphenylphosphine (1 g, 3.81 mmol, 3 eq) and water (0.5 mL) were added. The resulting mixture was stirred at ambient temperature for 20 h. The reaction mixture was filtered through an anhydrous Na_2_SO_4_ and then solvents were evaporated under reduced pressure. The residue was subjected to a silica gel column chromatography (MeOH/CH_2_Cl_2_ 1:9) to give *24-aminocholan-3α-ol* (**14**) (0.44 g, 97%) as a white solid. ^1^H NMR (400 MHz, methanol-d_4_) δ: 3.56 (m, 1H, H-3β), 2.62 (m, 2H, H-24), 0.94 (d, 3H, *J* = 6.4 Hz, H-21), 0.93 (s, 3H, H-19), 0.66 (s, 3H, H-18); ^13^C NMR (100 MHz, methanol-d_4_) δ: 71.6 (CH), 57.0 (CH), 56.6 (CH), 43.0 (C), 42.6 (CH), 42.3 (CH_2_), 40.9 (CH), 40.6 (CH_2_), 36.3 (CH_2_), 36.3 (CH), 36.0 (CH), 35.7 (CH_2_), 34.9 (C), 33.4 (CH_2_), 30.3 (CH_2_), 29.6 (CH_2_), 28.6 (CH_2_), 27.5 (CH_2_), 26.8 (CH_2_), 24.5 (CH_2_), 23.5 (CH_3_), 21.1 (CH_2_), 18.7 (CH_3_), 12.1 (CH_3_).

#### 3.2.2. Reaction of 24-aminocholan-3α-ol (**14**) with bromoacetonitrile

A solution of aminosteroid **14** (81 mg, 0.22 mmol) in absolute ethanol (10 mL) was treated with a drop of bromoacetonitrile (1.8 µL, 0.27 mmol, 1.2 equiv.) in presence of anhydrous sodium carbonate (28.5 mg, 0.27 mmol, 1.2 eq). The solution was stirred at 55 °C for 4 h, cooled to room temperature, concentrated under reduced pressure and purified by a silica gel column chromatography. *24-(N-Cyanomethyl)aminocholan-3α-ol* (**15a**) was obtained as a white solid in 61% (55 mg) yield using MeOH/CH_2_Cl_2_ (1:9) for elution. M.p. 111–113 °C (MeOH/CH_2_Cl_2_); ^1^H NMR (400 MHz, CDCl_3_) δ: 3.61 (m, 1H, H-3β), 3.59 (s, 2H, CH_2_-CN), 2.69 (m, 2H, H-24), 0.9129 (d, 3H, *J* = 6.4 Hz, H-21), 0.9133 (s, 3H, H-19), 0.64 (s, 3H, H-18); ^13^C NMR (100 MHz, CDCl_3_) δ: 117.8 (CN), 71.7 (CH), 56.5 (CH), 56.1 (CH), 49.4 (CH_2_), 42.6 (C), 42.0 (CH), 40.4 (CH), 40.1 (CH_2_), 37.3 (CH_2_), 36.4 (CH_2_), 35.8 (CH), 35.6 (CH), 35.3 (CH_2_), 34.5 (C), 33.2 (CH_2_), 30.5 (CH_2_), 28.3 (CH_2_), 27.1 (CH_2_), 26.4 (CH_2_), 26.0 (CH_2_), 24.2 (CH_2_), 23.3 (CH_3_), 20.8 (CH_2_), 18.6 (CH_3_), 12.0 (CH_3_); IR (ATR, cm^−1^) ν: 3311, 2926, 2857, 2238, 1454; HRMS calcd for C_26_H_45_N_2_O 401.3532 [M + H]^+^, found: 401.3530.

#### 3.2.3. Reaction of 24-aminocholan-3α-ol (**14**) with acrylonitrile 

Acrylonitrile (2.2 µL, 0.33 mmol, 1.5 eq) was added to a solution of 24-aminocholan-3α-ol 14 (80 mg, 0.22 mmol, 1.0 eq) in absolute ethanol (10 mL). The reaction mixture was then stirred at room temperature for 30 min and at reflux for 1 h, cooled to room temperature, then evaporated under reduced pressure to give the crude product. *24-(N-2′-Cyanoethyl)-aminocholan-3α-ol* (**16a**) was purified by a silica gel column chromatography with MeOH/CH_2_Cl_2_ (1:24) elution affording product as a white solid in 92% (85 mg) yield. M.p. 92–93 °C (MeOH/CH_2_Cl_2_); ^1^H NMR (400 MHz, CDCl_3_) δ: 3.60 (m, 1H, H-3β), 2.92 (t, 2H, *J* = 6.6 Hz, CH_2_-CN), 2.58 (m, 2H, H-24), 2.52 (t, 2H, *J* = 6.6 Hz, NH-CH_2_), 0.91 (s, 3H, H-19), 0.90 (d, 3H, *J* = 5.9 Hz, H-21), 0.63 (s, 3H, H-18); ^13^C NMR (100 MHz, CDCl_3_) δ: 118.6 (CN), 71.6 (CH), 56.4 (CH), 56.1 (CH), 49.7 (CH_2_), 45.0 (CH_2_), 42.6 (C), 42.0 (CH), 40.4 (CH), 40.1 (CH_2_), 36.4 (CH_2_), 35.8 (CH), 35.6 (CH), 35.3 (CH_2_), 34.5 (C), 33.3 (CH_2_), 30.5 (CH_2_), 28.3 (CH_2_), 27.1 (CH_2_), 26.5 (CH_2_), 26.4 (CH_2_), 24.1 (CH_2_), 23.3 (CH_3_), 20.7 (CH_2_), 18.6 (CH_2_), 18.6 (CH_3_), 11.9 (CH_3_); IR (ATR, cm^−1^) ν: 3301, 2926, 2856, 2246, 1453, 1368; HRMS calcd for C_27_H_47_N_2_O 415.3688, [M + H]^+^, found: 415.3892.

#### 3.2.4. Reduction of 24-(*N*-cyanomethyl)-aminocholan-3α-ol (**15a**) and 24-(*N*-2′-cyanoethyl)-aminocholan-3α-ol (**16a**)

To a solution of nitrile **15a** (40 mg, 0.10 mmol) in anhydrous diethyl ether (10 mL) was added lithium aluminum hydride (15 mg, 0.4 mmol, 4 eq). The reaction mixture was stirred at room temperature for 10 min and at reflux for 3 h. Then, the mixture was cooled to room temperature and water was added dropwise followed by 20% aqueous sodium hydroxide. The resultant slurry was stirred for 15 min and anhydrous Na_2_SO_4_ was added. The stirring was continued for an additional 15 min. Solid materials were washed with diethyl ether and removed by filtration. The filtrate was concentrated under reduced pressure. The crude product was purified by a silica gel column chromatography with MeOH/CH_2_Cl_2_/NH_3_aq (50:50:3) elution affording *24-*(*2′-amino-ethylamino)-cholan-3α-ol* (**17a**) as a white solid in 82% (33 mg) yield. M.p. 107–110 °C (MeOH); ^1^H NMR (400 MHz, methanol-d_4_) δ: 3.54 (m, 1H, H-3β), 2.79 (t, 2H, *J* = 5.9 Hz, N-CH_2_), 2.70 (t, 2H, *J* = 5.9 Hz, N-CH_2_), 2.58 (m, 2H, H-24), 0.97 (d, 3H, *J* = 6.5 Hz, H-21), 0.95 (s, 3H, H-19), 0.70 (s, 3H, H-18); ^13^C NMR (100 MHz, methanol-d4) δ: 72.4 (CH), 58.0 (CH), 57.6 (CH), 52.1 (CH_2_), 51.1 (CH_2_), 43.9 (C), 43.5 (CH), 41.9 (CH), 41.6 (CH_2_), 41.3 (CH_2_), 37.2 (CH), 37.18 (CH_2_), 37.1 (CH), 36.5 (CH_2_), 35.7 (C), 34.7 (CH_2_), 31.2 (CH_2_), 29.4 (CH_2_), 28.4 (CH_2_), 27.7 (CH_2_), 26.9 (CH_2_), 25.3 (CH_2_), 24.0 (CH_3_), 22.0 (CH_2_), 19.2 (CH_3_), 12.5 (CH_3_); IR (ATR, cm^−1^) ν: 3303, 2925, 2856, 1453, 1368; HRMS calcd for C_26_H_49_N_2_O 405.3845 [M + H]^+^, found: 405.3863.

*24-(3′-Amino-propylamino)-cholan-3α-ol* (**18a**) was prepared in a similar way from **16a** using MeOH/CH_2_Cl_2_/NH_3_aq (4:1:01) for elution. The pure product **18a** was obtained as a white solid in 80% (69 mg) yield. M.p. 92–94 °C (MeOH/CH_2_Cl_2_); ^1^H NMR (400 MHz, MeOD) δ: 3.54 (m, 1H, H-3β), 2.73 (t, 2H, *J* = 7.1 Hz, N-*C*H_2_), 2.67 (t, 2H, *J* = 7.3 Hz, N-CH_2_), 2.58 (m, 2H, H-24), 0.97 (d, 3H, *J* = 6.5 Hz, H-21), 0.95 (s, 3H, H-19), 0.69 (s, 3H, H-18); ^13^C NMR (100 MHz, methanol-d_4_) δ: 72.4 (CH), 58.0 (CH), 57.6 (CH), 51.1 (CH_2_), 48.2 (CH_2_), 43.9 (C), 43.5 (CH), 41.9 (CH), 41.6 (CH_2_), 40.5 (CH_2_), 37.2 (CH), 37.18 (CH_2_), 37.1 (CH), 36.5 (CH_2_), 35.7 (C), 34.7 (CH_2_), 32.2 (CH_2_), 31.2 (CH_2_), 29.4 (CH_2_), 28.4 (CH_2_), 27.7 (CH_2_), 26.7 (CH_2_), 25.3 (CH_2_), 23.9 (CH_3_), 22.0 (CH_2_), 19.2 (CH_3_), 12.5 (CH_3_); IR (ATR, cm^−1^) ν: 3272, 2925, 2856, 1453, 1367; HRMS calcd for C_27_H_51_N_2_O 419.4001 [M + H]^+^, found: 419.4068.

#### 3.2.5. Esterification of bile acids **7a**–**7d**

A solution of lithocholic acid **7a** (3 g, 8 mmol) in methanol (200 mL) was treated with two drops of concentrated hydrochloric acid and the mixture was stirred at ambient temperature for 48 h. After this time, sodium bicarbonate was added to neutralize the reaction mixture and the solvent was evaporated. The residue was extracted with methylene chloride (3 × 100 mL) and dried over anhydrous sodium sulfate. The organic solvent was evaporated and the residue was filtered through a silica gel column with AcOEt/hexane (3:17) as an eluent. The pure *methyl lithocholate* (**19a**) was obtained as a white solid in 96% (3 g) yield. ^1^H NMR (400 MHz, CDCl_3_) δ: 3.66 (s, 3H, O-CH_3_), 3.62 (m, 1H, H-3β), 2.35 and 2.22 (each m, 1H and 1H, H-23), 0.92 (s, 3H, H-19), 0.91 (d, 3H, *J* = 6.1 Hz, H-21), 0.64 (s, 3H, H-18); ^13^C NMR (100 MHz, CDCl_3_) δ: 174.7 (C=O), 71.5 (CH), 56.3 (CH), 55.8 (CH), 51.3 (CH_3_), 42.6 (C), 41.9 (CH), 40.3 (CH), 40.0 (CH_2_), 36.2 (CH_2_), 35.7 (CH), 35.24 (CH_2_), 35.2 (CH), 34.4 (C), 30.9 (CH_2_), 30.8 (CH_2_), 30.3 (CH_2_), 28.0 (CH_2_), 27.1 (CH_2_), 26.3 (CH_2_), 24.0 (CH_2_), 23.2 (CH_3_), 20.7 (CH_2_), 18.1 (CH_3_), 11.9 (CH_3_).

*Methyl deoxycholate* (**19b**) was prepared in a similar way from deoxycholic acid using MeOH/CH_2_Cl_2_ (1:19) for elution. The pure product **19b** was obtained as a white solid in 98% (1.52 g) yield. ^1^H NMR (400 MHz, CDCl_3_) δ: 3.98 (m, 1H, H-12β), 3.68 (s, 3H, O-CH_3_), 3.61 (m, 1H, H-3β), 2.38 and 2.24 (each m, 1H and 1H, H-23), 0.97 (d, 3H, *J* = 6.3 Hz, H-21), 0.91 (s, 3H, H-19), 0.68 (s, 3H, H-18); ^13^C NMR (100 MHz, CDCl_3_) δ: 174.7 (C=O), 73.0 (CH), 71.6 (CH), 51.4 (CH_3_), 48.1 (CH), 47.1 (CH), 46.4 (C), 42.0 (CH), 36.3 (CH_2_), 35.9 (CH), 35.2 (CH_2_), 35.16 (CH), 34.0 (C), 33.5 (CH), 31.1 (CH_2_), 30.8 (CH_2_), 30.3 (CH_2_), 28.5 (CH_2_), 27.4 (CH_2_), 27.1 (CH_2_), 26.1 (CH_2_), 23.6 (CH_2_), 23.1 (CH_3_), 17.2 (CH_3_), 12.6 (CH_3_).

*Methyl hyodeoxycholate* (**19c**) was prepared in a similar way from hyodeoxycholic acid with MeOH/CH_2_Cl_2_ (1:19) elution. The product **19c** was obtained as a white solid in 97% (5 g) yield. ^1^H NMR (400 MHz, CDCl_3_) δ: 4.07 (m, 1H, H-6β), 3.68 (s, 3H, O-CH_3_), 3.61 (m, 1H, H-3β), 2.37 and 2.23 (each m, 1H and 1H, H-23), 0.924 (d, 3H, *J* = 6.3 Hz, H-21), 0.918 (s, 3H, H-19), 0.65 (s, 3H, H-18); ^13^C NMR (100 MHz, CDCl_3_) δ: 174.6 (C=O), 71.3 (CH), 67.8 (CH), 56.1 (CH), 55.9 (CH), 51.4 (CH_3_), 48.4 (CH), 42.7 (C), 39.9 (CH_2_), 39.8 (CH), 35.8 (C), 35.6 (CH_2_), 35.3 (CH), 34.7 (CH), 34.6 (CH_2_), 31.0 (CH_2_), 30.8 (CH_2_), 29.9 (CH_2_), 29.1 (CH_2_), 28.0 (CH_2_), 24.1 (CH_2_), 23.5 (CH_3_), 20.7 (CH_2_), 18.1 (CH_3_), 11.9 (CH_3_).

*Methyl cholate* (**19d**) was obtained in a similar way from cholic acid as a white solid in 91% (4.6 g) yield using AcOEt/hexane (9:1) for elution. ^1^H NMR (400 MHz, CDCl_3_) δ: 3.99 (m, 1H, H-12β), 3.87 (m, 1H, H-7β), 3.68 (s, 3H, -O-CH_3_), 3.47 (m, 1H, H-3β), 0.99 (d, 3H, *J* = 6.3 Hz, H-21), 0.91 (s, 3H, H-19), 0.70 (s, 3H, H-18); ^13^C NMR (100 MHz, CDCl_3_) δ: 174.7 (C=O), 72.8 (CH), 71.6 (CH), 68.1 (CH), 51.3 (CH_3_), 46.7 (CH), 46.2 (C), 41.33 (CH), 41.3 (CH), 39.3 (CH), 39.1 (CH_2_), 35.2 (CH), 35.1 (CH_2_), 34.6 (CH_2_), 34.6 (C), 30.9 (CH_2_), 30.7 (CH_2_), 30.2 (CH_2_), 28.0 (CH_2_), 27.4 (CH_2_), 26.0 (CH), 23.0 (CH_2_), 22.3 (CH_3_), 17.1 (CH_3_), 12.2 (CH_3_).

#### 3.2.6. Reaction of methyl esters **19a**–**d** with ethylenediamine

Methyl lithocholate (**19a**) (1 g, 2.6 mmol) was dissolved in methanol (5 mL), then ethylenediamine (1.55 mL, 23.4 mmol, 9 eq) was added. The reaction mixture was stirred at 65 °C for 96 h. Methanol and the lion’s share of ethylenediamine were removed under reduced pressure. *N-(2′-Aminoethyl)-lithocholamide* (**20a**) was purified by a silica gel column chromatography with MeOH/NH_3_aq (20:0.07) elution affording product as a white solid in 90% (0.96 g) yield. M.p. 172–174 °C (MeOH); ^1^H NMR (400 MHz, methanol-d_4_) δ: 3.54 (m, 1H, H-3β), 3.23 (t, 2H, *J* = 6.4 Hz, CO-NH-CH_2_), 2.71 (t, 2H, *J* = 6.4 Hz, CH_2_-NH_2_), 2.26 and 2.11 (each m, 1H and 1H, CH_2_-CO-NH), 0.97 (d, 3H, *J* = 6.5 Hz, H-21), 0.95 (s, 3H, H-19), 0.69 (s, 3H, H-18); ^13^C NMR (100 MHz, methanol-d_4_) δ: 177.1 (C=O), 72.4 (CH), 57.9 (CH), 57.4 (CH), 43.9 (C), 43.5 (CH), 43.0 (CH_2_), 42.0 (CH_2_), 41.9 (CH), 41.5 (CH_2_), 37.2 (CH), 37.18 (CH_2_), 36.9 (CH), 36.5 (CH_2_), 35.7 (C), 34.1 (CH_2_), 33.3 (CH_2_), 31.2 (CH_2_), 29.3 (CH_2_), 28.4 (CH_2_), 27.7 (CH_2_), 25.3 (CH_2_), 24.0 (CH_3_), 22.0 (CH_2_), 18.9 (CH_3_), 12.5 (CH_3_); IR (ATR, cm^−1^) ν: 3340, 3266, 3080, 2925, 2857, 1649, 1552; HRMS calcd for C_26_H_47_N_2_O_2_ 419.3638 [M + H]^+^, found: 419.3621.

*N-(2′-Aminoethyl)-deoxycholamide* (**20b**) was purified by a silica gel column chromatography with MeOH/NH_3_aq (40:0.07) elution affording product as a white solid in 90% (0.48 g) yield. M.p. 110–112 °C (MeOH); ^1^H NMR (400 MHz, methanol-d_4_) δ: 3.96 (m, 1H, H-12β), 3.52 (m, 1H, H-3β), 3.23 (t, 2H, *J* = 6.4 Hz, CO-NH-CH_2_), 2.71 (t, 2H, *J* = 6.4 Hz, CH_2_-NH_2_), 2.26 and 2.13 (each m, 1H and 1H, CH_2_-CO-NH), 1.02 (d, 3H, *J* = 6.3 Hz, H-21), 0.93 (s, 3H, H-19), 0.71 (s, 3H, H-18); ^13^C NMR (100 MHz, methanol-d_4_) δ: 177.2 (C=O), 74.0 (CH), 72.5 (CH), 49.3 (CH), 48.1 (CH), 47.6 (C), 43.6 (CH), 42.9 (CH_2_), 42.0 (CH_2_), 37.5 (CH), 37.2 (CH_2_), 36.9 (CH), 36.4 (CH_2_), 35.3 (C), 34.8 (CH), 34.1 (CH_2_), 33.3 (CH_2_), 31.1 (CH_2_), 29.9 (CH_2_), 28.7 (CH_2_), 28.4 (CH_2_), 27.5 (CH_2_), 24.9 (CH_2_), 23.7 (CH_3_), 17.7 (CH_3_), 13.2 (CH_3_); IR (ATR, cm^−1^) ν: 3293, 2923, 2862, 1633, 1544; HRMS calcd for C_26_H_47_N_2_O_3_ 435.3587 [M + H]^+^, found: 435.3579.

*N-(2′-Aminoethyl)-hyodeoxycholamide* (**20c**) was purified by a silica gel column chromatography with MeOH/NH_3_aq (20:0.07) elution affording product as a white solid in 87% (0.93 g) yield. M.p. 102–104 °C (MeOH); ^1^H NMR (400 MHz, methanol-d_4_) δ: 4.01 (m, 1H, H-6β), 3.51 (m, 1H, H-3β), 3.23 (t, 2H, *J* = 6.4 Hz, CO-NH-CH_2_), 2.71 (t, 2H, *J* = 6.4 Hz, CH_2_-NH_2_), 2.26 and 2.11 (each m, 1H and 1H, CH_2_-CO-NH), 0.97 (d, 3H, *J* = 6.5 Hz, H-21), 0.93 (s, 3H, H-19), 0.69 (s, 3H, H-18); ^13^C NMR (100 MHz, methanol-d_4_) δ: 177.1 (C=O), 72.4 (CH), 68.6 (CH), 57.6 (CH), 57.4 (CH), 49.9 (CH), 44.0 (C), 43.0 (CH_2_), 42.0 (CH_2_), 41.4 (CH_2_), 41.3 (CH), 36.9 (CH), 36.86 (CH_2_), 36.8 (C), 36.2 (CH), 35.6 (CH_2_), 34.1 (CH_2_), 33.2 (CH_2_), 31.1 (CH_2_), 30.0 (CH_2_), 29.2 (CH_2_), 25.3 (CH_2_), 24.1 (CH_3_), 21.9 (CH_2_), 18.9 (CH_3_), 12.5 (CH_3_); IR (ATR, cm^−1^) ν: 3281, 3085, 2928, 2860, 1643, 1546; HRMS calcd for C_26_H_47_N_2_O_3_ 435.3587 [M + H]^+^, found: 435.3577.

*N-(2′-Aminoethyl)-cholamide* (**20d**) was purified by a silica gel column chromatography with MeOH/NH_3_aq (100:0.9) elution affording product as a white solid in 92% (0.98 g) yield. M.p. 135–137 °C (MeOH/H_2_O); ^1^H NMR (400 MHz, methanol-d_4_) δ: 3.95 (m, 1H, H-12β), 3.80 (m, 1H, H-7β), 3.37 (m, 1H, H-3β), 3.24 (t, 2H, *J* = 6.3 Hz, CO-NH-CH_2_), 2.73 (t, 2H. *J* = 6.3 Hz, CH_2_-NH_2_), 1.03 (d, 3H, *J* = 6.3 Hz, H-21), 0.92 (s, 3H, H-19), 0.71 (s, 3H, H-18); ^13^C NMR (100 MHz, methanol-d_4_) δ: 177.3 (C=O), 74.0 (CH), 72.9 (CH), 69.0 (CH), 48.0 (CH), 47.5 (C), 43.2 (CH), 43.0 (CH), 42.8 (CH_2_), 42.0 (CH_2_), 41.0 (CH), 40.5 (CH_2_), 36.9 (CH), 36.5 (CH_2_), 35.9 (CH_2_), 35.9 (C), 34.1 (CH_2_), 33.3 (CH_2_), 31.2 (CH_2_), 29.6 (CH_2_), 28.7 (CH_2_), 27.9 (CH), 24.2 (CH_2_), 23.2 (CH_3_), 17.7 (CH_3_), 13.0 (CH_3_); IR (ATR, cm^−1^) ν: 3348, 3289, 2924, 2864, 1642, 1547; HRMS calcd for C_26_H_47_N_2_O_4_ 451.3536 [M + H]^+^, found: 451.3567.

#### 3.2.7. Reaction of methyl esters **19a**–**d** with propane-1,3-diamine

Methyl lithocholate (**19a**) (0.8 g, 2 mmol) was dissolved in methanol (4 mL), then propane-1,3-diamine (1.2 mL, 14 mmol, 7 eq) was added. The reaction mixture was stirred at 65 °C for 72 h. The solvent and the lion’s share of propane-1,3-diamine were removed under reduced pressure. The crude product was purified by a silica gel column chromatography. Elution with MeOH/NH_3_aq (20:0.07) afforded *N-(3′-aminopropyl)-lithocholamide* (**21a**) as a white solid in 82% (0.73 g) yield. M.p. 73–75 °C (MeOH); ^1^H NMR (400 MHz, methanol-d_4_) δ: 3.54 (m, 1H, H-3β), 3.23 (t, 2H, *J* = 6.8 Hz, CO-NH-CH_2_), 2.65 (t, 2H, *J* = 7.0 Hz, CH_2_-NH_2_), 2.23 and 2.10 (each m, 1H and 1H, CH_2_-CO-NH), 0.96 (d, 3H, *J* = 6.6 Hz, H-21), 0.95 (s, 3H, H-19), 0.69 (s, 3H, H-18); ^13^C NMR (100 MHz, methanol-d_4_) δ: 176.9 (C=O), 72.4 (CH), 57.9 (CH), 57.4 (CH), 43.9 (C), 43.5 (CH), 41.9 (CH), 41.5 (CH_2_), 39.7 (CH_2_), 37.6 (CH_2_), 37.2 (CH), 37.2 (CH_2_), 36.9 (CH), 36.5 (CH_2_), 35.7 (C), 34.1 (CH_2_), 33.4 (CH_2_), 33.37 (CH_2_), 31.2 (CH_2_), 29.3 (CH_2_), 28.4 (CH_2_), 27.7 (CH_2_), 25.3 (CH_2_), 23.9 (CH_3_), 21.9 (CH_2_), 18.9 (CH_3_), 12.5 (CH_3_); IR (ATR, cm^−1^) ν: 3351, 2927, 2857, 1638, 1550; HRMS calcd for C_27_H_49_N_2_O_2_ 433.3794 [M + H]^+^, found: 433.3785.

*N-(3′-Aminopropyl)-deoxycholamide* (**21b**) was purified by a silica gel column chromatography with MeOH/NH_3_aq (20:0.07) elution affording product as a white solid in 85% (0.47 g) yield. M.p. 130–132 °C (MeOH); ^1^H NMR (400 MHz, methanol-d_4_) δ: 3.96 (m, 1H, H-12β), 3.52 (m, 1H, H-3β), 3.23 (t, 2H, *J* = 6.8 Hz, CO-NH-CH_2_), 2.65 (t, 2H, *J* = 7.0 Hz, CH_2_-NH_2_), 2.25 and 2.10 (each m, 1H and 1H, CH_2_-CO-NH), 1.02 (d, 3H, *J* = 6.4 Hz, H-21), 0.93 (s, 3H, H-19), 0.71 (s, 3H, H-18); ^13^C NMR (100 MHz, methanol-d_4_) δ: 177.0 (C=O), 74.0 (CH), 72.5 (CH), 49.3 (CH), 48.1 (CH), 47.6 (C), 43.6 (CH), 39.7 (CH_2_), 37.6 (CH_2_), 37.5 (CH), 37.2 (CH_2_), 36.9 (CH), 36.4 (CH_2_), 35.3 (C), 34.8 (CH), 34.1 (CH_2_), 33.4 (CH_2_), 33.3 (CH_2_), 31.1 (CH_2_), 29.9 (CH_2_), 28.7 (CH_2_), 28.4 (CH_2_), 27.5 (CH_2_), 24.9 (CH_2_), 23.7 (CH_3_), 17.7 (CH_3_), 13.2 (CH_3_); IR (ATR, cm^−1^) ν: 3295, 2921, 2860, 1634, 1546; HRMS calcd for C_27_H_49_N_2_O_3_ 449.3743 [M + H]^+^, found: 449.3804.

*N-(3′-Aminopropyl)-hyodeoxycholamide* (**21c**) was purified by a silica gel column chromatography. Elution with MeOH/NH_3_aq (20:0.07) afforded product as a white solid in 89% (0.98 g) yield. M.p. 89–91 °C (MeOH); ^1^H NMR (400 MHz, methanol-d_4_) δ: 4.01 (m, 1H, H-6β), 3.50 (m, 1H, H-3β), 3.23 (t, 2H, *J* = 6.8 Hz, CO-NH-CH_2_), 2.65 (t, 2H, *J* = 7.0 Hz, CH_2_-NH_2_), 2.23 and 2.10 (each m, 1H and 1H, CH_2_-CO-NH), 0.97 (d, 3H, *J* = 6.5 Hz, H-21), 0.93 (s, 3H, H-19), 0.69 (s, 3H, H-18); ^13^C NMR (100 MHz, methanol-d_4_) δ: 176.9 (C=O), 72.4 (CH), 68.6 (CH), 57.6 (CH), 57.4 (CH), 49.9 (CH), 44.0 (C), 41.4 (CH_2_), 41.3 (CH), 39.7 (CH_2_), 37.6 (CH_2_), 36.9 (C), 36.85 (CH), 36.8 (CH_2_), 36.2 (CH), 35.6 (CH_2_), 34.1 (CH_2_), 33.4 (CH_2_), 33.3 (CH_2_), 31.1 (CH_2_), 30.0 (CH_2_), 29.2 (CH_2_), 25.3 (CH_2_), 24.1 (CH_3_), 21.9 (CH_2_), 18.8 (CH_3_), 12.5 (CH_3_); IR (ATR, cm^−1^) ν: 3282, 2930, 2864, 1642, 1543; HRMS calcd for C_27_H_49_N_2_O_3_ 449.3743 [M + H]^+^, found: 449.3758. 

*N-(3′-Aminopropyl)-cholamide* (**21d**) was purified by a silica gel column chromatography with MeOH/NH_3_aq (20:0.07) elution affording product as a white solid in 89% (0.49 g) yield. M.p. 98–101 °C (AcOEt/MeOH); ^1^H NMR (400 MHz, methanol-d_4_) δ: 3.95 (m, 1H, H-12β), 3.80 (m, 1H, H-7β), 3.37 (m, 1H, H-3β), 3.23 (t, 2H, *J* = 6.8 Hz, CO-NH-CH_2_), 2.68 (t, 2H, *J* = 7.0 Hz, CH_2_-NH_2_), 1.03 (d, 3H, *J* = 6.3 Hz, H-21), 0.92 (s, 3H, H-19), 0.71 (s, 3H, H-18); ^13^C NMR (100 MHz, methanol-d_4_) δ: 177.1 (C=O), 74.0 (CH), 72.9 (CH), 69.0 (CH), 48.0 (CH), 47.5 (C), 43.2 (CH), 43.0 (CH), 41.0 (CH), 40.5 (CH_2_), 39.5 (CH_2_), 37.5 (CH_2_), 36.9 (CH), 36.5 (CH_2_), 35.9 (CH_2_), 35.9 (C), 34.1 (CH_2_), 33.4 (CH_2_), 32.9 (CH_2_), 31.2 (CH_2_), 29.6 (CH_2_), 28.7 (CH_2_), 27.9 (CH), 24.2 (CH_2_), 23.2 (CH_3_), 17.7 (CH_3_), 13.0 (CH_3_); IR (ATR, cm^−1^) ν: 3344, 3208, 3093, 2925, 2862, 1625, 1561; HRMS calcd for C_27_H_49_N_2_O_4_ 465.3692 [M + H]^+^, found: 465.3672.

#### 3.2.8. Reduction of amides **20a**–**d** and **21a**–**d**

To a cooled (4 °C) suspension of *N*-(2-aminoethyl)-lithocholamide (**20a**) (0.5 g, 1.2 mmol) in anhydrous THF (30 mL) lithium aluminum hydride (228 mg, 6 mmol, 5 eq) was added. The reaction mixture was stirred at reflux for 72 h. Then it was cooled to room temperature and water (0.1 mL) was added dropwise, followed by 20% aqueous sodium hydroxide (0.2 mL). The resultant slurry was stirred for 15 min, and then anhydrous sodium sulfate was added. Stirring was continued for an additional 15 min. Solid materials were removed by filtration, washed with THF and the filtrate concentrated under reduced pressure.

This general work-up procedure cannot be applied for hyodeoxycholic acid amide derivatives (**20c** or **21c**) due to an easy degradation of products under described conditions. In these cases a routine extractive work-up (decomposition of hydride with a large excess of water and extraction with dichloromethane) was used.

*24-*(*2′-Amino-ethylamino)-cholan-3α-ol* (**17a**) was purified by a silica gel column chromatography with MeOH/CH_2_Cl_2_/NH_3_aq (50:50:3) elution affording product as a white solid in 68% (0.33 g) yield. 

*24-(3′-Amino-propylamino)-cholan-3α-ol* (**18a**) was purified by a silica gel column chromatography with MeOH/CH_2_Cl_2_/NH_3_aq (4:1:01) elution affording product as a white solid in 62% (0.15 g) yield. 

*24-(2′-Amino-ethylamino)-cholane-3α,12α-diol* (**17b**) was purified by a silica gel column chromatography with MeOH/NH_3_aq (20:0.07) elution affording product as a white solid in 67% (65 mg) yield. M.p. 70–72 °C (MeOH); ^1^H NMR (400 MHz, methanol-d_4_) δ: 3.96 (m, 1H, H-12β), 3.52 (m, 1H, H-3β), 2.80 (t, 2H, *J* = 5.9 Hz, N-CH_2_), 2.71 (t, 2H, *J* = 5.9 Hz, N-CH_2_), 2.59 (m, 2H, H-24), 1.03 (d, 3H, *J* = 6.5 Hz, H-21), 0.93 (s, 3H, H-19), 0.71 (s, 3H, H-18); ^13^C NMR (100 MHz, methanol-d4) δ: 74.1 (CH), 72.6 (CH), 51.9 (CH_2_), 51.1 (CH_2_), 49.8 (CH), 48.3 (CH), 47.5 (C), 43.6 (CH), 41.2 (CH_2_), 37.5 (CH), 37.2 (CH_2_), 37.1 (CH), 36.4 (CH_2_), 35.3 (C), 34.8 (CH), 34.7 (CH_2_), 31.1 (CH_2_), 29.9 (CH_2_), 28.8 (CH_2_), 28.4 (CH_2_), 27.5 (CH_2_), 26.9 (CH_2_), 24.9 (CH_2_), 23.7 (CH_3_), 18.0 (CH_3_), 13.2 (CH_3_); IR (ATR, cm^−1^) ν: 3287, 2924, 2858, 1452, 1369; HRMS calcd for C_26_H_49_N_2_O_2_ 421.3794 [M + H]^+^, found: 421.3794.

*24-(3′-Amino-propylamino)-cholane-3α,12α-diol* (**18b**) was purified by a silica column chromatography with MeOH/NH_3_aq (100:1) elution affording product as a white solid in 65% (63 mg) yield. M.p. 64–66 °C (MeOH); ^1^H NMR (400 MHz, methanol-d_4_) δ: 3.96 (m, 1H, H-12β), 3.52 (m, 1H, H-3β), 2.69 (t, 2H, *J* = 7.0 Hz, N-CH_2_), 2.64 (t, 2H, *J* = 7.4 Hz, N-CH_2_), 2.56 (m, 2H, H-24), 1.02 (d, 3H, *J* = 6.4 Hz, H-21), 0.93 (s, 3H, H-19), 0.71 (s, 3H, H-18); ^13^C NMR (100 MHz, methanol-d_4_) δ: 74.1 (CH), 72.6 (CH), 51.3 (CH_2_), 49.9 (CH), 48.3 (CH), 48.26 (CH_2_), 47.5 (C), 43.6 (CH), 40.6 (CH_2_), 37.5 (CH), 37.2 (CH_2_), 37.1 (CH), 36.4 (CH_2_), 35.3 (C), 34.8 (CH), 34.7 (CH_2_), 32.9 (CH_2_), 31.1 (CH_2_), 29.9 (CH_2_), 28.8 (CH_2_), 28.4 (CH_2_), 27.5 (CH_2_), 27.0 (CH_2_), 24.9 (CH_2_), 23.7 (CH_3_), 18.0 (CH_3_), 13.2 (CH_3_); IR (ATR, cm^−1^) ν: 3278, 2925, 2858, 1454, 1369; HRMS calcd for C_27_H_51_N_2_O_2_ 435.3951 [M + H]^+^, found: 435.3946.

*24-(2′-Amino-ethylamino)-cholane-3α,6α-diol* (**17c**) was purified by a silica gel column chromatography with MeOH/NH_3_aq (25:0.2) elution affording product as a white solid in 69% (167 mg) yield. M.p. 72–74 °C (MeOH); ^1^H NMR (400 MHz, methanol-d_4_) δ: 4.01 (m, 1H, H-6β), 3.51 (m, 1H, H-3β), 2.75 (t, 2H, *J* = 5.9 Hz, N-CH_2_), 2.65 (t, 2H, *J* = 5.9 Hz, N-CH_2_), 2.55 (m, 2H, H-24), 0.97 (d, 3H, *J* = 6.5 Hz, H-21), 0.93 (s, 3H, H-19), 0.70 (s, 3H, H-18); ^13^C NMR (100 MHz, methanol-d_4_) δ: 72.4 (CH), 68.6 (CH), 57.7 (CH), 57.6 (CH), 52.7 (CH_2_), 51.3 (CH_2_), 49.9 (CH), 44.0 (C), 41.7 (CH_2_), 41.4 (CH_2_), 41.3 (CH), 37.1 (CH), 36.9 (C), 36.8 (CH_2_), 36.2 (CH), 35.6 (CH_2_), 34.7 (CH_2_), 31.1 (CH_2_), 30.0 (CH_2_), 29.3 (CH_2_), 27.1 (CH_2_), 25.3 (CH_2_), 24.1 (CH_3_), 21.9 (CH_2_), 19.2 (CH_3_), 12.5 (CH_3_); IR (ATR, cm^−1^) ν: 3285, 2927, 2857, 1453, 1368; HRMS calcd for C_26_H_49_N_2_O_2_ 421.3794 [M + H]^+^, found: 421.4040.

*24-(3′-Amino-propylamino)-cholane-3α,6α-diol***18c** was purified by a silica gel column chromatography with MeOH/NH_3_aq (100:1) elution affording product as a white solid in 73% (106 mg) yield. M.p. 64–66 °C (MeOH); ^1^H NMR (400 MHz, methanol-d_4_) δ: 4.01 (m, 1H, H-6β), 3.51 (m, 1H, H-3β), 2.72 (t, 2H, *J* = 7.1 Hz, N-CH_2_), 2.66 (t, 2H, *J* = 7.3 Hz, N-CH_2_), 2.57 (m, 2H, H-24), 0.97 (d, 3H, *J* = 6.4 Hz, H-21), 0.93 (s, 3H, H-19), 0.69 (s, 3H, H-18); ^13^C NMR (100 MHz, methanol-d_4_) δ: 72.4 (CH), 68.6 (CH), 57.7 (CH), 57.5 (CH), 51.2 (CH_2_), 49.9 (CH), 48.2 (CH_2_), 44.0 (C), 41.4 (CH_2_), 41.3 (CH), 40.5 (CH_2_), 37.1 (CH), 36.9 (C), 36.8 (CH_2_), 36.2 (CH), 35.6 (CH_2_), 34.7 (CH_2_), 32.4 (CH_2_), 31.1 (CH_2_), 30.0 (CH_2_), 29.3 (CH_2_), 26.8 (CH_2_), 25.3 (CH_2_), 24.1 (CH_3_), 21.9 (CH_2_), 19.2 (CH_3_), 12.4 (CH_3_); IR (ATR, cm^−1^) ν: 3275, 2926, 2858, 1454, 1369; HRMS calcd for C_27_H_51_N_2_O_2_ 435.3951 [M + H]^+^, found: 435.3927. 

*24-(2′-Amino-ethylamino)-cholane-3α,7α,12α-triol***17d** was purified by a silica gel column chromatography with MeOH/ CH_2_Cl_2_/NH_3_aq (5:5:0.7) elution affording product as a white solid in 67% (0.32 g) yield. M.p. 107–111 °C (MeOH); ^1^H NMR (400 MHz, methanol-d_4_) δ: 3.96 (m, 1H, H-12β), 3.80 (m, 1H, H-7β), 3.37 (m, 1H, H-3β), 2.77 (t, 2H, *J* = 5.9 Hz, N-CH_2_), 2.68 (t, 2H, *J* = 6.1 Hz, N-CH_2_), 2.57 (m, 2H, H-24), 1.03 (d, 3H, *J* = 6.5 Hz, H-21), 0.92 (s, 3H, H-19), 0.72 (s, 3H, H-18); ^13^C NMR (100 MHz, methanol-d_4_) δ: 74.1 (CH), 72.9 (CH), 69.1 (CH), 52.4 (CH_2_), 51.2 (CH_2_), 48.3 (CH), 47.5 (C), 43.2 (CH), 43.0 (CH), 41.5 (CH_2_), 41.0 (CH), 40.5 (CH_2_), 37.2 (CH), 36.5 (CH_2_), 35.9 (C), 35.88 (CH_2_), 34.7 (CH_2_), 31.2 (CH_2_), 29.6 (CH_2_), 28.8 (CH_2_), 27.9 (CH), 27.1 (CH_2_), 24.2 (CH_2_), 23.2 (CH_3_), 18.0 (CH_3_), 13.0 (CH_3_); IR (ATR, cm^−1^) ν: 3328, 2925, 2854, 1467, 1369; HRMS calcd for C_26_H_49_N_2_O_3_ 437.3743 [M + H]^+^, found: 437.3765.

*24-(3′-Amino-propylamino)-cholane-3α,7α,12α-triol***18d** was purified by a silica gel column chromatography with MeOH/CH_2_Cl_2_/NH_3_aq (5:5:1) elution affording product as a white solid in 65% (0.63 g) yield. M.p. 105–108 °C (MeOH); ^1^H NMR (400 MHz, methanol-d_4_) δ: 3.96 (m, 1H, H-12β), 3.80 (m, 1H, H-7β), 3.37 (m, 1H, H-3β), 2.79 (t, 2H, *J* = 7.1 Hz, N-CH_2_), 2.75 (t, 2H, *J* = 7.3 Hz, N-CH_2_), 2.65 (m, 2H, H-24), 1.04 (d, 3H, *J* = 6.5 Hz, H-21), 0.92 (s, 3H, H-19), 0.72 (s, 3H, H-18); ^13^C NMR (100 MHz, methanol-d_4_) δ: 74.0 (CH), 72.9 (CH), 69.0 (CH), 50.9 (CH_2_), 48.2 (CH), 48.0 (CH_2_), 47.4 (C), 43.2 (CH), 43.0 (CH), 41.0 (CH), 40.5 (CH_2_), 40.3 (CH_2_), 37.1 (CH), 36.5 (CH_2_), 35.9 (CH_2_), 35.9 (C), 34.6 (CH_2_), 31.2 (CH_2_), 31.0 (CH_2_), 29.6 (CH_2_), 28.8 (CH_2_), 27.9 (CH), 26.4 (CH_2_), 24.2 (CH_2_), 23.2 (CH_3_), 18.0 (CH_3_), 13.0 (CH_3_); IR (ATR, cm^−1^) ν: 3352, 2925, 2860, 1454, 1371; HRMS calcd for C_27_H_51_N_2_O_3_ 451.3900 [M + H]^+^, found: 451.3932.

#### 3.2.9. General procedure for preparation of complexes from K_2_PtCl_4_ and bile acid-derived diamines **17a**–**d** and **18a**–**d**

The solution of potassium tetrachloroplatinate (51 mg, 0.12 mmol) in DMF (1.5 mL) and distilled water (1.5 mL) were added to a solution of 24-(2-amino-ethylamino)-cholan-3α-ol (**17a**) (50 mg, 0.12 mmol) in DMF (1.5 mL). The resulting mixture was stirred in the dark for 3 days. Then, the solvent was evaporated and the residue was stirred vigorously in a saturated aqueous potassium chloride solution (4 mL) for 20 min. The resulting suspension was filtered, washed with water, and dried in a desiccator over phosphorus pentoxide for 1 day. *cis-Dichloro-24-[(N-2′-aminoeth-1′-yl-κN)aminocholan-3α-ol-κN]platinum(II)* (**22a**) was obtained in 76% (63 mg) yield. IR (ATR, cm^−1^) ν: 3445, 3197, 3122, 2926, 2857, 1585, 1451, 1371, 1028; ^195^Pt NMR (86 MHz, DMF) δ: -2336.6; HRMS calcd for C_26_H_48_Cl_2_N_2_O^196^Pt [M + Na]^+^: 693.2703 found: 693.2654.

*cis-Dichloro-24-[(N-3′-aminoprop-1′-yl-κN)aminocholan-3α-ol-κN]platinum(II)* (**23a**) was prepared in a similar way. The product **23a** was obtained in 80% (65 mg) yield. IR (ATR, cm^−1^) ν: 3473, 3223, 3091, 2926, 2857, 1605, 1450, 1372, 1030; ^195^Pt NMR (86 MHz, DMF) δ: −2264.3; HRMS calcd for C_27_H_50_Cl_2_N_2_O^196^Pt [M + Na]^+^: 707.2860 found: 707.2840.

*cis-Dichloro-24-[(N-2′-aminoeth-1′-yl-κN)aminocholane-3α,12α-diol-κN]platinum(II*) (**22b**) was prepared in a similar way. The product **22b** was obtained in 67 % (54 mg) yield. IR (ATR, cm^−1^) ν: 3425, 3196, 3124, 2926, 2861, 1651, 1452, 1374, 1032; ^195^Pt NMR (86 MHz, DMF) δ: −2335.2; HRMS calcd for C_26_H_48_Cl_2_N_2_O_2_^196^Pt [M + Na]^+^: 709.2652 found: 709.2644.

*cis-Dichloro-24-[(N-3′-aminoprop-1′-yl-κN)aminocholane-3α,12α-diol-κN]platinum(II)* (**23b**) was prepared in a similar way. The product **23b** was obtained in 78% (62 mg) yield. IR (ATR, cm^−1^) ν: 3420, 3196, 3147, 2924, 2860, 1595, 1446, 1375, 1039; ^195^Pt NMR (86 MHz, DMF) δ: −2265.6; HRMS calcd for C_27_H_50_Cl_2_N_2_O_2_^196^Pt [M + Na]^+^: 723.2809 found: 723.2785.

*cis-Dichloro-24-[(N-2′-aminoeth-1′-yl-κN)aminocholane-3α,6α-diol-κN]platinum(II)* (**22c**) was prepared in a similar way. The product **22c** was obtained in 74% (60 mg) yield. IR (ATR, cm^−1^) ν: 3393, 3198, 3133, 2929, 2860, 1627, 1453, 1373, 1031; ^195^Pt NMR (86 MHz, DMF) δ: −2336.1; HRMS calcd for C_26_H_48_Cl_2_N_2_O_2_^196^Pt [M + Na]^+^: 709.2652 found: 709.2644.

*cis-Dichloro-24-[(N-3′-aminoprop-1′-yl-κN)aminocholane-3α,6α-diol-κN]platinum(II)* (**23c**) was prepared in a similar way. The product **23c** was obtained in 69% (55 mg) yield. IR (ATR, cm^−1^) ν: 3423, 3205, 3140, 2929, 2860, 1594, 1452, 1373, 1034; ^195^Pt NMR (86 MHz, DMF) δ: −2264.6; HRMS calcd for C_27_H_50_Cl_2_N_2_O_2_^196^Pt [M + Na]^+^: 723.2809 found: 723.2785.

*cis-Dichloro-24-[(N-2′-aminoeth-1′-yl-κN)aminocholane-3α,7α,12α-triol-κN]platinum(II*) (**22d**) was prepared in a similar way. The product **22d** was obtained in 81% (65 mg) yield. IR (ATR, cm^−1^) ν: 3424, 3203, 3126, 2922, 2861, 1586, 1454, 1372, 1030; ^195^Pt NMR (86 MHz, DMF) δ: −2334.9; HRMS calcd for C_26_H_48_Cl_2_N_2_O_3_^196^Pt [M + Na]^+^: 725.2601 found: 725.2579.

*cis-Dichloro-24-[(N-3′-aminoprop-1′-yl-κN)aminocholane-3α,7α,12α-triol-κN]platinum(II)* (**23d**) was prepared in a similar way. The product **23d** was obtained in 61% (48 mg) yield. IR (ATR, cm^−1^) ν: 3412, 3211, 3138, 2926, 2863, 1604, 1453, 1374, 1035; ^195^Pt NMR (86 MHz, DMF) δ: −2257.8; HRMS calcd for C_27_H_50_Cl_2_N_2_O_3_^196^Pt [M + Na]^+^: 739.2758 found: 739.2723.

### 3.3. Cytotoxicity Assay

Cisplatin derivatives were evaluated for their cytotoxic activities *in vitro* in following cell lines derived from various tumors: T-lymphoblastic leukemia (CEM), epithelial breast carcinoma (MCF7) and cervical carcinoma cell lines (HeLa) (all from ECACC, UK) and also in normal human fibroblasts (BJ; from ATCC, USA). Human umbilical vein endothelial cells (HUVEC) were a kind gift from Prof Jitka Ulrichová (Medical Faculty, Palacký University, Olomouc, Czech Republic). HUVEC were grown in ECPM medium (Provitro, Berlin, Germany). Six serial 3-fold dilutions in triplicate of each drug in DMF was administered in every cell line for 72 h. Alamar Blue dye was used for staining of living cells only. IC_50_ values (50% inhibitory concentrations) were calculated as the proportions of surviving cells. A detailed procedure for the cytotoxicity assay was described previously, except for using Alamar Blue dye instead of Calcein AM [37]. Briefly, the percentage of surviving cells in each well was calculated by dividing the intensity of the fluorescence signals from the exposed wells by the intensity of signals from control wells and multiplying by 100. These ratios were then used to construct dose–response curves, from which IC_50_ values, i.e., concentrations of the respective compounds that were lethal to 50% of the tumor cells, were calculated. The results obtained from cytotoxicity assays are shown in Table 2 (mean values ± SD obtained from three independent experiments performed in triplicate).

## 4. Conclusions

In summary, we have developed two new synthetic routes to the derivatives of bile acids possessing 1,2- and 1,3-diamino moieties in the side chain. In the first synthesis the key transformation was bromoacetonitrile alkylation of the primary amine or its addition to acrylonitrile. The crucial step of the second strategy was monoamidation of a methyl ester with diamine. These methodologies provide simple, convenient and efficient routes to the synthesis of various steroidal diamines. Comparison of these strategies, taking as an example transformation of lithocholic acid (**7**) into diamines **17a** and **18a**, has proved that the second, significantly shorter synthetic route affords the final products in higher (**17a**) or similar (**18a**) yield. The described herein protocol for the preparation of diamines can be also applied in a large scale synthesis. The platinum complexes obtained from the diamines showed a moderate cytotoxicity but their selectivity against human cancer cells was unsatisfactory.

## Figures and Tables

**Table 1:** Starting bile acids, their synthesized derivatives and target platinum(II) complexes.

Acids	Esters	Amides	Amines	Complexes
Lithocholic acid 7**a** (R_1_=R_2_=R_3_=H)	**19a**	n = 2, **20a** n = 3, **21a**	n = 2, **17a** n = 3, **18a**	n = 1, **22a** n = 2, **23a**
Deoxycholic acid **7b** (R_1_=R_2_=H; R_3_=OH)	**19b**	n = 2, **20b** n = 3, **21b**	n = 2, **17b** n = 3, **18b**	n = 1, **22b** n = 2, **23b**
Hyodeoxycholic acid **7c** (R_1_=OH; R_2_=R_3_=H)	**19c**	n = 2, **20c** n = 3, **21c**	n = 2, **17c** n = 3, **18c**	n = 1, **22c** n = 2, **23c**
Cholic acid **7d** (R_1_=H; R_2_=R_3_=OH)	**19d**	n = 2, **20d** n = 3, **21d**	n = 2, **17d** n = 3, **18d**	n = 1, **22d** n = 2, **23d**

**Table 2:** IC_50_ (µM, in DMF) values ± S.D. for the Pt(II)-complexes after 72 h of treatment.

Complexes	Cells IC_50_ (µM)				
	CEM	MCF7	HeLa	BJ	HUVEC
**22a** *	40.2 ± 2.6	>50	>50	>50	>50
**23a**	3.6 ± 0.1	7.0 ± 0.5	7.0 ± 0.2	6.3 ± 0.8	6.9 ± 0.6
**22b**	37.1 ± 6.8	20.5 ± 3.1	35.5 ± 5.3	36.6 ± 5.1	38.2 ± 0.6
**23b**	31.6 ± 3.5	23.3 ± 4.7	36.2 ± 4.3	42.5 ± 3.2	34.4 ± 5.6
**6** [7]	>50	>50	28.0 ± 4.7	>50	>50
**cisplatin**	0.8 ± 0.1	7.7 ± 1.7	11.4 ± 3.8	6.9 ± 0.9	5.5 ± 1.1

* test done at temperature 37 °C due to solubility problems.
